# Prevalence of Bacterial Febrile Illnesses in Children in Kilosa District, Tanzania

**DOI:** 10.1371/journal.pntd.0003750

**Published:** 2015-05-08

**Authors:** Beatrice Chipwaza, Ginethon G. Mhamphi, Steve D. Ngatunga, Majige Selemani, Mbaraka Amuri, Joseph P. Mugasa, Paul S. Gwakisa

**Affiliations:** 1 Nelson Mandela African Institution of Science and Technology, Arusha, Tanzania; 2 Ifakara Health Institute, Ifakara, Tanzania; 3 Sokoine University of Agriculture, Pest Management Center, Chuo Kikuu, Morogoro, Tanzania; 4 National Institute for Medical Research, Morogoro, Tanzania; 5 Ifakara Health Institute, Dar-es-Salaam, Tanzania; 6 Jhpiego, Dar-es-Salaam, Tanzania; 7 National Institute for Medical Research, Amani Medical Research Centre, Tanga, Tanzania; 8 Genome Science Centre and Department of Veterinary Microbiology and Parasitology, Sokoine University of Agriculture, Morogoro, Tanzania; University of Tennessee, UNITED STATES

## Abstract

**Introduction:**

Bacterial etiologies of non-malaria febrile illnesses have significantly become important due to high mortality and morbidity, particularly in children. Despite their importance, there are few reports on the epidemiology of these diseases in Tanzania, and the true burden of such illnesses remains unknown. This study aimed to identify the prevalence of leptospirosis, brucellosis, typhoid fever and urinary tract infections and their rate of co-infections with malaria.

**Methods:**

A cross-sectional study was conducted at Kilosa district hospital in Tanzania for 6 months. Febrile children aged from 2–13 years were recruited from the outpatient department. Patients were screened by serological tests such as IgM and IgG ELISA, and microscopic agglutination test.

**Results:**

A total of 370 patients were enrolled; of these 85 (23.0%) had malaria parasites, 43 (11.6%) had presumptive acute leptospirosis and 26/200 (13%) had confirmed leptospirosis. Presumptive acute brucellosis due to *B*. *abortus* was identified among 26 (7.0%) of patients while *B*. *melitensis* was detected in 57 (15.4%) of the enrolled patients. Presumptive typhoid fever due to *S*. Typhi was identified in thirty eight (10.3%) of the participants and 69 (18.6%) had urinary tract infections. Patients presented with similar symptoms; therefore, the identification of these diseases could not be done based on clinical ground alone. Co-infections between malaria and bacterial febrile illnesses were observed in 146 patients (39.5%). Although antibacterials and/or anti-malarials were prescribed in most patients, some patients did not receive the appropriate treatment.

**Conclusion:**

The study has underscored the importance of febrile bacterial diseases including zoonoses such as leptospirosis and brucellosis in febrile children, and thus such illnesses should be considered by clinicians in the differential diagnoses of febrile diseases. However, access to diagnostic tests for discrimination of febrile illnesses is needed. This would allow febrile patients to receive the correct diagnoses and facilitation of accurate and prompt treatment.

## Author Summary

Understanding the bacterial causes of fever, particularly in the era of reported decline of malaria, is very important. In Tanzania, data on the prevalence of these diseases in several regions is limited. These infections cannot be recognized clinically, and thus the lack of diagnostic tools in most health facilities leads to misdiagnosis and mistreatment of febrile patients. This cross-sectional study was carried out in children at Kilosa district hospital in Tanzania. Among 370 enrolled patients, 85 (23.0%) had malaria parasites, 43 (11.6%) had presumptive acute leptospirosis and 26/200 (13%) had confirmed leptospirosis. Presumptive acute brucellosis due to *B*. *abortus* was identified in 26 (7.0%) and *B*. *melitensis* was among 57 (15.4%) of the enrolled patients. Presumptive typhoid fever was observed in 38 patients (10.3%), while 69 (18.6%) had urinary tract infections. Co-infections between bacterial diseases with malaria were observed and thus indicate the necessity of considering multiple infections in patients with malaria. These findings are of relevance to clinicians since they should consider bacterial causes of fever including zoonotic diseases in the routine diagnosis of febrile patients. Also, the findings provide evidence to policy makers and national disease control programs.

## Introduction

In recent years, there has been a progressive decline in malaria transmission and morbidity which in line with a decline in proportions of fevers caused by malaria therefore many fever cases are likely to be non-malaria [1]. Febrile illnesses due to bacterial etiologies contribute significantly to morbidity and mortality particularly in children in developing countries, including Tanzania [2, 3]. The bacterial diseases such as urinary tract infections (UTI), acute respiratory tract infections and typhoid fever have been reported as common causes of fever in children in Tanzania [4, 5]. Urinary tract infections refer to the presence of bacterial pathogens within the urinary tract (bladder or kidney). The most common pathogen associated with UTIs is *Escherichia coli* which accounts for 80% of the isolates [6]. With regards to acute respiratory tract, a recent study reported a prevalence of 59 (88%) in children all due to *Streptococcus pneumonia* [4]. Salmonella enterica serotype Typhi (*S*. Typhi) and non-Typhi have been demonstrated as important causes of typhoid fever in Tanzanian children [7]. Typhoid fever is both waterborne and foodborne and therefore poor access to safe water, sanitation and hygiene infrastructure are the major risk factors.

Furthermore, bacterial zoonotic diseases such leptospirosis and brucellosis have also been reported [3]. Leptospirosis is caused by spirochaetes of the genus *Leptospira* with several different serovars [8]. The disease is spread by the urine of infected animals and thus close human contact with livestock or wild animals, and poor sanitation are among the predisposing factors. Human can get infection through direct contact with the urine of infected animals or by contact with a urine-contaminated environment, such as surface water, soil and plants. Human brucellosis is caused by species of *Brucella*, the most common are *B*. *abortus* and *B*. *melitensis*. *Brucella abortus* is mostly associated with cattle while *B*. *melitensis* is typically associated with sheep and goats. The infection is transmitted to humans by ingestion of infectious animal products such as milk, dairy products or meat, direct contact with infected animals and their products through skin abrasions or conjunctiva and inhalation of airborne particulates [9].

The diagnosis of bacterial febrile illnesses poses a challenge particularly in resource poor countries where laboratory diagnostic facilities are limited [10]. Most of bacterial febrile illnesses could have similar symptoms with malaria and hence making it difficult to distinguish such illnesses clinically. Similarly, differential diagnosis of several bacterial febrile illnesses without laboratory tests is difficult due to overlapping clinical manifestations among these diseases. In Tanzania, the lack of diagnostic facilities in most health facilities have led to rely on clinical diagnosis and thus these diseases are unrecognized or being underreported. Apart from few studies conducted in northern Tanzania, the true burden of such illnesses remains poorly undefined in many parts of the country [11, 12, 13]. In addition, despite the reported decline of malaria, many febrile patients are still presumed to have malaria and thus malaria over diagnosis is commonly encountered and contribute to improper treatment of febrile patients [10]. Furthermore, non-malaria febrile illnesses cases which occur concurrently with malaria are often not recognized and thus treatment of patients with anti-malarial alone results into incomplete therapy. This study aimed at determining the prevalence of bacterial febrile illnesses in children in Kilosa district. Moreover, the study identified co-infections between bacterial febrile illnesses and malaria as well as co-infections between bacterial febrile illnesses.

## Materials and Methods

### Study setting

**Fig 1:**
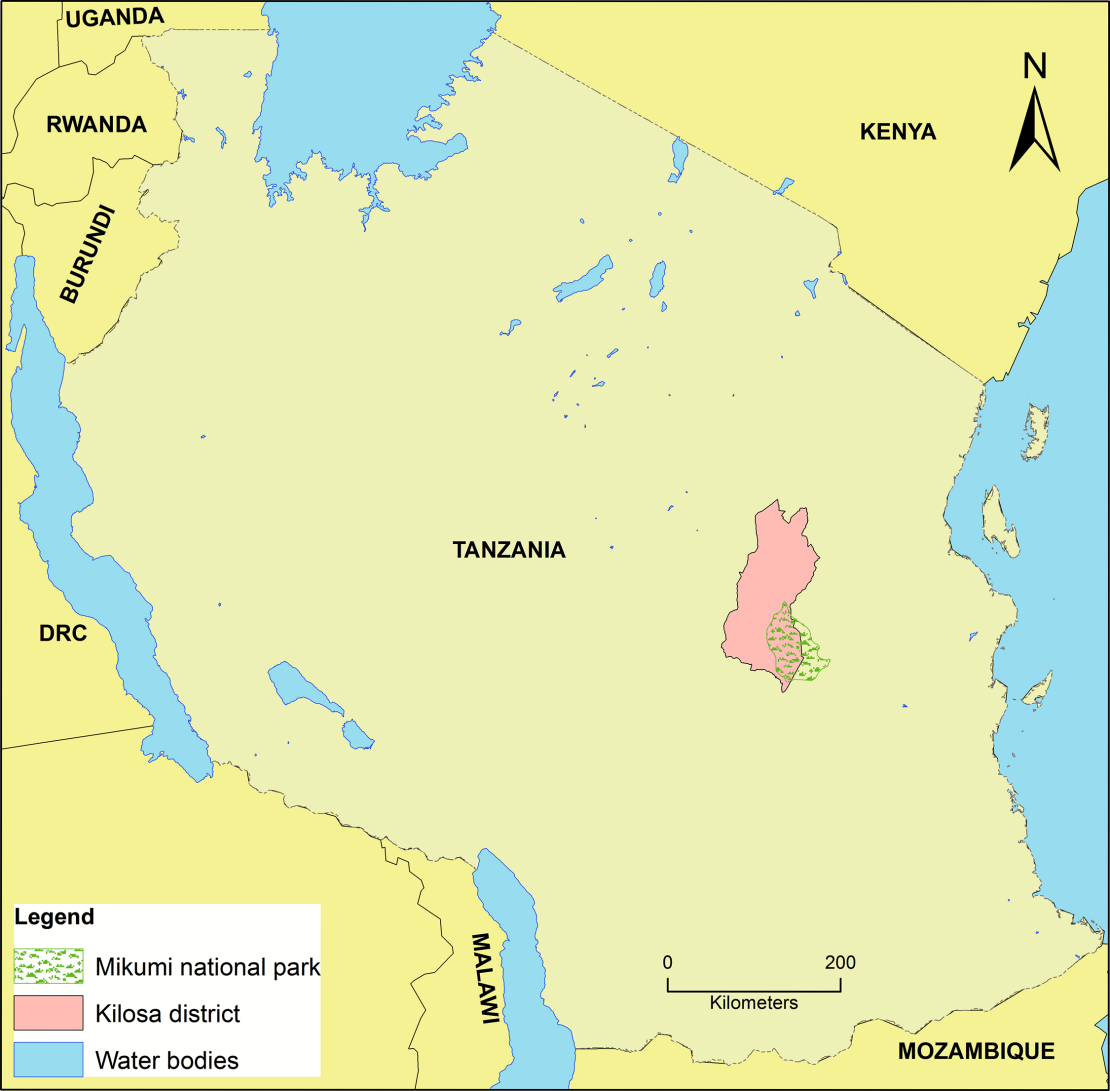
Map of Tanzania showing the study area.

The study was conducted at Kilosa district hospital which is situated in Morogoro region, Tanzania (Fig 1). The district lies between latitudes 6° south and 8° south and longitudes 36° 30’ east and 38° east. The climate typically consists of short rain period which starts in November and end in January followed by heavy rainfall between March and May. The district experiences dry season from June to October. The average annual temperature is 24.6°C. The district has an area of 14,245 square kilometers and the population is 438,175 people [14]. The number of children less than 5 years of age and 5–10 years is 65,654 and 62,235 respectively [15]. The main economic activities are crop production and livestock keeping. Kilosa district is divided into 9 divisions, 37 wards and 164 villages [16]. In addition, there are 71 health facilities, of which 3 are hospitals, 7 health centers and 61 dispensaries [17]. Kilosa district hospital serves as referral hospital for the primary health care facilities in the district [18].

Kilosa was selected due to its possession of intensive human activities with livestock and its proximity to wildlife from the Mikumi National Park (Fig 1). Therefore, the district has a good interface for zoonotic diseases such as brucellosis and leptospirosis. Kilosa district is an area with holoendemic malaria transmission with seasonal peaks following the long and short rainy seasons [19]. In 2007–2008 malaria prevalence was estimated to be 15.7% in Morogoro region [20] and decreased to 13% in the year 2011–2012 [21]. Non-malaria febrile illnesses that have been reported in Kilosa district include acute respiratory diseases, UTIs and typhoid fever [22]. Data from a platform for health monitoring and evaluation in Tanzania (Sentinel Panel of Districts) have shown that in the year 2011, acute respiratory diseases and UTIs comprised of 20% and 2.5% respectively of total recorded illnesses (77,862) in outpatient department in children aged less than 5 years [23]. According to 2005/2006 statistics, the infant mortality rate and under-five mortality rate in Kilosa district were 112 per 1000 live births and 166 per 1000 live births respectively [24] while in 2012/2013 the rates were 74 and 192 per 1000 live births respectively [25].

### Study participants and procedures

This study was conducted for 6 months, during rainy season (March—May 2013) and dry season (August—October 2013). The participants were recruited from febrile children who attended outpatient department at Kilosa district hospital. The inclusion criteria were children aged from 2–13 years, axillary temperature ≥37.5°C or rectal temperature ≥ 38.0°C at the time of recruitment. Fever was classified as mild if temperature was 37.5–38.3°C, moderate at 38.4–39.4°C and high at >39.5°C. Children with chronic diseases or those who required immediate attention were excluded. The estimation of the sample size for the assessment of prevalence of febrile illnesses in study area was based on 36%, which is a reported prevalence of malaria and other febrile febrile illnesses such as acute respiratory tract infections and UTIs [10]. The assumptions for the confidence level and margin of error were 95% (1.96) and 5% (0.05) respectively while contingencies such as recording error was 5%. According to Naing *et al*. (2006) the sample size (n) was calculated as n = (1.96)^2^ (0.36)(1–0.36)/(0.05)^2^ = 354; Total n = 354 + 17.7 = 372 [26].

A trained clinical officer from the hospital collected demographic information, obtained clinical history and performed physical examination for each enrolled participant. The clinical diagnoses and treatment were also performed by the respective clinician and were recorded on a standard assessment form. Blood specimen 5ml of venous blood was collected aseptically from each febrile patient in plain tubes and thereafter serum was separated by centrifugation. In addition urine sample was collected from each patient in sterile tubes and processed immediately. Serum samples were temporarily stored at—20°C at Kilosa district hospital before being transported to Ifakara Health Institute (IHI) where they were kept at–80°C until analyzed.

### Laboratory methods

Laboratory investigation was done based on selected bacterial febrile illnesses that might occur within the district. Thus, the selected bacterial febrile illnesses were leptospirosis, brucellosis, urinary tract infection and typhoid fever. In addition, all patients were screened for malaria parasites. Viral causes of fever were also investigated and detailed results have been presented in a separate paper.

#### Malaria

The presence of malaria parasites was checked by microscopy by a laboratory technician from the respective hospital. Thick and thin blood films were prepared and stained with Giemsa and examined for blood parasites. Each slide was read independently by a second experienced microscopist from IHI and any discrepancies were resolved by a third microscopist. The parasite density was determined by standard methods [27].

#### Leptospirosis

Laboratory diagnosis of leptospirosis was done by the detection of IgM and IgG antibodies in serum by using enzyme-linked immunosorbent assay (ELISA) at IHI, Ifakara, Tanzania. ELISA kits were obtained from Serion Immundiagnostica GmbH (Germany) and the ELISA procedure was carried out as per manufacturer’s instructions. The ELISA kits contain extract from *Leptospira biflexa* containing genus-specific epitopes directed against all *Leptospira* species. Each set of tests was run with positive and negative controls. The test was valid when the absorbance reading of the above meets the specification of the manufacturer’s and the results were interpreted according to the manufacturer’s recommendation. The sensitivity and specificity of these tests, as determined by the manufacturers, are shown in S1 Table. Positive specimens were subsequently tested by microscopic agglutination test (MAT) which is considered the gold standard for leptospirosis serodiagnosis [8]. This test was conducted at the Pest Management Center in Sokoine University of Agriculture, Morogoro, Tanzania. The MAT test was performed using standard procedure [8]. Six *Leptospira* serovars were used; this includes Kenya, Lora, Sokoine and Grippotyhosa (local isolates from domestic animals and rodents) and other serovars were *Leptospira* reference strains i.e. Hardjo and Hebdomadis. Briefly, live *Leptospira* cell suspensions were incubated with serially diluted serum specimens in U- bottomed 96-well plates. Negative and positive controls for each serovar were included. The plates with serum–antigen mixture were incubated at 30°C for 2–4 hours before being examined for agglutination under dark field microscopy. To confirm the agglutination titer, serum samples that showed agglutination were further diluted to up to 1: 20,480. The reported titer was the highest dilution of serum that agglutinated at least 50% of the cells for each serovar tested [8]. In our study, a single specimen (acute serum) was used and thus a cut-off titer of ≥ 1:160 was considered an indicative of acute infection [28, 29, 30]. Presumptive acute leptospisosis was defined as a positive IgM antibodies result for *Leptospira* while probable prior leptospirosis exposure was defined as a positive anti-*Leptospira* IgG ELISA result. Confirmed acute leptospirosis was defined as a positive MAT result.

#### Brucellosis

NovaLisa™ *Brucella* IgM-ELISA and NovaLisa™ *Brucella* IgG-ELISA were used according to the manufacturer’s instructions to detect the presence of *Brucella abortus*-specific IgM and IgG antibodies in serum samples (NovaTec Immundiagnostica GmbH, Germany). The kits contained microtiter strips pre-coated with a lysate antigen of *B*. *abortus*. The recommended cutoff values were used to determine negative, positive and borderline results, however, all border line results were considered negative. The sensitivity and specificity of these tests, as determined by the manufacturers, are shown in S1 Table. All serum samples were also screened by a stained *Brucella* suspension for detection of antibodies toward *Brucella abortus* and *Brucella melitensis* (Span Diagnostics Ltd, India). The rapid screening test was done by adding one drop of a well-shaken antigen suspension (*B*. *abortus or B*. *melitensis*) on a slide containing 20 μl of undiluted serum. Serum samples which showed a visible agglutination within 1 minute were quantified by tube agglutination test. For the tube method, the serum samples were diluted serially from 1:20 to 1:1280 followed by the addition of a single drop of antigen into the respective tubes. A negative control (saline) was also included. The tubes were incubated at 37°C for 16–20 hours. The positivity cut-off point for tube agglutination reaction was defined as antibody titers ≥ 1:160 [31]. Presumptive acute brucellosis was defined as a positive IgM antibodies result for *B*. *abortus* and probable prior brucellosis exposure was defined as a positive anti-*Brucella* IgG ELISA result.

#### Typhoid fever

The detection of antibodies toward *Salmonella* Typhi was done by a Widal test which was based on agglutination reaction between *S*. Typhi somatic lipopolysacharide O antigens and flagellar H antigens [32]. This test was performed with standardized O and H antigens (Agappe Diagnostics, Switzerland). All suspensions were stored at 2–8°C in the dark and then thawed to room temperature before use. The initial test done was the rapid screening test, whereby one drop of undiluted serum was placed in a 3cm diameter circle on a white tile and a drop of the O and H antisera was added and spread over the entire circle. The results were read after immediate rotation of the slide for 1 minute. Positive sera were further tested by tube method as described in the section under brucellosis. The positivity cut-off point for tube agglutination reaction for both O and H antigens was defined as antibody titers ≥ 1:160 [32] and was considered as presumptive typhoid fever.

#### Urinary tract infections

Urine samples were examined visually and microscopically. Briefly, microscopic examination was performed by centrifugation of urine at 3000 rpm for 3 minutes and thereafter the supernatant was discarded aseptically. The sediment was placed on the slide and a cover slip was applied before being examined under the microscope at 40X objective. An average count of white blood cells (WBC) was taken per number of fields examined. The increased number of leucocytes is mostly observed in urinary tract diseases, and thus presence of ≥5 WBCs per hpf (high-power field) was considered positive [33].

### Data management and statistical analysis

Data were entered into an Access database by an experienced data clerk. The database was designed to ascertain validation rules of each data field. The verification of data entry and data cleaning was done to ensure that clinical data and laboratory findings are matched. The cleaned data were then transferred to STATA using Stat transfer version 9 and statistical analyses were performed using STATA software (version 11; Stata Corp., TX USA). The outcome variables were bacterial febrile illnesses i.e. leptospirosis, brucellosis, urinary tract infection, typhoid fever and malaria. Pearson’s Chi-Square test was used to determine the association between categorical variables. An alpha level of 0.05 was used for all tests of statistical significance. In addition, logistic regression model for multivariate analysis was used to assess the relationship between the selected bacterial febrile illnesses and sex, age and season. Interpretation of results was based on the odds ratio and 95% confidence intervals.

### Ethics statement

This study was approved by Institutional Review Board of Ifakara Health Institute (IHI/IRB/No: 01–2013) and Medical Research Coordinating Committee of Tanzania’s National Institute for Medical Research (NIMR/HQ/R.8a/Vol.1X/1472). Children with less than 12 years had written informed consent given from a parent or guardian while children over 12 years provided their own written informed consent which was accompanied by a written consent of a parent or guardian. In addition a verbal assent was also obtained from children aged 7–12 years.

## Results

A total of 370 febrile children were enrolled, of these 189 (51.1%) were males and 181 (48.8%) were females. Among the enrolled patients, 205 (55.4%) were children aged less than five years and 165 (44.6%) were aged five years and above (Table 1). One hundred and eighty nine patients (51.1%) were recruited during the rainy season while 181 (48.9) were enrolled in the dry season. At the time of enrollment, the majority of patients (75.7%; n = 280) had mild fever whereas 85 (23.0%) and 5 (1.3%) had moderate and high fever respectively. In addition, 75 (20.3%) of patients reported prior use of anti-malarials while 11 (2.9%) had used antibiotics and 18 (4.9%) had previously used both anti-malarials and antibiotics. The demographic characteristics of patients as well as enrollment characteristics are summarized in Table 1.

**Table 1:** Patients’ demographic and enrollment characteristics (N = 370).

	Characteristic	n (%)
**Gender**	Male	189 (51.1)
	Female	181 (48.9)
**Age**	< 5 years	205 (55.4)
	≥5 years	165 (44.6)
**Season**	Wet season	189 (51.1)
	Dry season	181 (48.9)
**Temperature**	Mild fever	280 (75.7)
	Moderate fever	85 (23.0)
	High fever	5 (1.3)
**Recent therapy**	No recent therapy	266 (71.9)
	Anti-malarials	75 (20.3)
	Antibacterials	11 (2.9)
	Anti-malarials & antibiotics	18 (4.9)

### Malaria

Among all 370 participants, 85 (23.0%) were positive for malaria parasites (*Plasmodium falciparum*), of these, 45/189 (23.8%) were males and 40/181 (22.1%) were females. Of 85 patients who had malaria, 47/205 (22.9%) were children aged less than 5 years while 38/165 (23.0%) were aged five years and above (Table 2). Although the difference in the occurrence of malaria cases between the two seasons was not statistically significant (OR = 1.53, 95% CI: 0.92–2.53), more cases of malaria were observed during the dry season 26.5% versus 19.6% (Table 2). The main diagnosis provided by the physician were UTI 25 (29.4%), pneumonia 3 (3.5%) and other infections 1 (1.2%), Table 2. With regards to treatment, 85(100%) of patients with malaria were treated with anti-malarials while 34 (40%) were treated with antibacterials, 74 (87.1%) with antipyretics and 1 (1.2%) were prescribed with other drugs (Table 2).

**Table 2:** Prevalence of malaria and bacterial febrile illnesses, the diagnosis and the prescribed treatment.

Subcategory	Malaria n/N (%)	Presumptive acute leptospirosis n/N (%)	Probable prior leptospirosis n/N (%)	Confirmed leptospirosis n/N (%)	Presumptive acute brucellosis n/N (%)	Probable prior brucellosis n/N (%)	*B*. *abortus* n/N (%)	*B*. *melitensis* n/N (%)	PresumptiveTyphoid fever n/N (%)
**Prevalence**	85/370 (23.0)	43/370 (11.6)	16/370 (4.3)	26/200 (13)	26/370 (7.0)	42/370 (11.4)	26/370 (7.0)	57/370 (15.4)	38/370 (10.3)
**Male**	45/189 (23.8)	16/189 (8.5)	10/189 (5.3)	17/89 (19.1)	13/189 (6.9)	13/189 (6.9)	13/189 (6.9)	22/189 (11.6)	13/189 (6.9)
**Female**	40/181 (22.1)	27/181 (14.9)	6/181 (3.3)	9/111 (8.1)	13/181 (7.2)	29/181 (16.0)	13/181 (7.2)	35/181 (19.3)	25/181 (13.8)
**< 5 years**	47/205 (22.9)	16/205 (7.8)	9/205 (4.4)	13/111 (11.7)	6/205 (2.9)	13/205 (6.3)	12/205 (5.9)	9/205 (4.4)	14/205 (6.8)
**≥ 5 years**	38/165 (23.0)	27/165 (16.4)	7/165 (4.2)	13/89 (14.6)	20/165 (12.1)	29/165 (17.6)	14/165 (8.5)	48/165 (29.1)	24/165 (14.5)
**Wet season**	37/189 (19.6)	30/189 (15.9)	16/189 (8.5)	14/84 (16.7)	19/189 (10.1)	28/189 (14.8)	18/189 (9.5)	38/189 (20.1)	20/189 (10.6)
**Dry season**	48/181 (26.5)	13/181 (7.2)	0	12/116 (10.3)	7/181 (3.9)	14/181 (7.7)	8/181 (4.4)	19/181 (10.5)	18/181 (9.9)
**Symptoms**									
Diarrhea	5/85 (5.9)	1/43 (2.3)	1/16 (6.3)	0	2/26 (7.7)	4/42 (9.5)	4/26 (15.4)	0	4/38 (10.5)
Vomiting	30/85 (35.3)	8/43 (18.6)	3/16 (18.8)	5/26 (19.2)	4/26 (15.4)	8/42 (19.1)	4/26 (15.4)	6/57 (10.5)	9/38 (23.7)
Loss of appetite	14/85 (16.5)	3/43 (7.0)	4/16 (25.0)	2/26 (7.7)	5/26 (19.2)	10/42 (23.8)	4/26 (15.4)	5/57 (8.8)	3/38 (7.9)
Joint pain	3/85 (3.5)	3/43 (7.0)	0	1/26 (3.8)	1/26 (3.9)	8/42 (19.0)	7/26 (26.9)	4/57 (7.0)	1/38 (2.6)
Abdominal pain	15/85 (17.7)	6/43 (14.0)	5/16 (31.3)	3/26 (11.5)	4/26 (15.4)	3/42 (7.1)	1/26 (3.9)	10/57 (17.5)	9/38 (23.7)
Headache	8/85 (9.4)	10/43 (23.3)	1/16 (6.3)	7/26 (26.9)	2/26 (7.7)	4/42 (9.5)	3/26 (11.5)	5/57 (8.8)	2/38 (5.3)
Other symptoms*	5/85 (5.9)	7/43 (16.3)	2/16 (12.5)	6/26 (23.1)	3/26 (11.5)	4/42 (9.5)	2/26 (7.7)	8/57 (14.0)	2/38 (5.3)
**Diagnoses**									
Malaria	85/85 (100)	13/43 (30.2)	2/16 (12.5)	7/26 (26.9)	3/26 (11.5)	8/42 (19.0)	3/26 (11.5)	9/57 (15.8)	13/38 (34.2)
UTI	25/85 (29.4)	22/43 (51.2)	9/16 (56.3)	11/26 (42.3)	14/26 (53.8)	21/42 (50.0)	9/26 (34.6)	35/57 (61.4)	17/38 (44.7)
Pneumonia	3/85 (3.5)	5/43 (11.6)	1/16 (6.3)	2/26 (7.7)	6/26 (23.1)	7/42 (16.7)	7/26 (26.9)	5/57 (8.8)	2/38 (5.3)
URT^§^	0	2/43 (4.7)	5/16 (31.3)	0	1/26 (3.9)	0	1/26 (3.8)	2/57 (3.5)	2/38 (5.3)
Other infections^#^	1/85 (1.2)	7/43 (16.3)	0	6/26 (23.1)	5/26 (19.2)	7/42 (16.7)	4/26 (15.4)	10/57 (17.5)	5/38 (13.2)
**Treatment**									
Anti-malarials	85/85 (100)	13/43 (30.2)	2/16 (12.5)	7/26 (26.9)	3/26 (11.5)	7/42 (16.7)	3/26 (11.5)	9/57 (15.8)	13/38 (34.2)
Antibacterials	34/85 (40)	35/43 (81.4)	15/16 (93.8)	21/26 (80.8)	25/26 (96.2)	35/42 (83.3)	24/26 (92.3)	51/57 (89.5)	31/38 (81.6)
Antipyretics	74/85 (87.1)	31/43 (72.1)	9/16 (56.3)	17/26 (65.4)	17/26 (65.4)	27/42 (64.3)	19/26 (73.1)	42/57 (73.7)	31/38 (81.6)
Other drugs^¶^	1/85 (1.2)	5/43 (11.6)	1/16 (6.3)	5/26 (19.2)	5/26 (19.2)	11/42 (26.2)	3/26 (11.5)	8/57 (14.0)	7/38 (18.4)

* Nasal discharge, cough, skin rashes, swollen tonsils, ear pain (discharge) and muscle aches;

^§^ UTR = Upper respiratory tract infections;

^**#**^ Chicken pox, boils, otitis media, enteric fever, herpes simplex, rhinitis and dysentery;

^**¶**^ Paediatric zinc, prednisolone, vitamins, ORS (oral rehydration salts) and antihelmintics.

### Leptospirosis

Among all the participants, 43 (11.6%) met the definition for presumptive acute leptospirosis (Table 2). Female patients were represented with a higher percentage 27/181 (14.9%) than males 16/189 (8.5%), (OR = 1.91, 95% CI: 0.98–3.75), Table 3. Despite the lack of statistical significance in age groups, a higher prevalence of leptospirosis was obtained in children older than 5 years of age (OR = 1.87, 95% CI: 0.95–3.68). Furthermore, few cases were detected during the dry season than in the rainy season (OR = 0.42, 95% CI: 0.21–0.86). Two hundred patients were tested by MAT, of these, 26 (13%) had confirmed leptospirosis with the following *Leptospira* serogroups; Sokoine 9 (34.6%), Kenya 6 (23.1%), Gripotyphosa 6 (23.1), Hebdomadis 6 (23.1%) and Lora 2 (7.7%). However, 2 patients had two *Leptospira* serogroups; one had Sokoine and Hebdomadis and the other patient had Sokoine and Gripotyphosa. Contrary to presumptive acute leptospirosis, female patients were less likely confirmed with leptospirosis (OR = 0.37, 95% CI: 0.15–0.88) as compared to males. Probable prior exposure to leptospirosis was observed in 16 (4.3%) of enrolled patients, of these, 10/189 (5.3%) were males and 6/181 (3.3%) were females and all cases occurred during the rainy season (Table 2). The main diagnosis in patients with presumptive acute leptospirosis was malaria 13 (30.2%) and UTIs 22 (51.2%). Also, the most common diagnoses in patients with confirmed leptospirosis were malaria 7 (26.9%) and UTIs 11 (42.3%). Most patients with presumptive acute leptospirosis and confirmed leptospirosis were treated with antibacterials and anti-malarials (Table 2). The most prescribed antibiotics were co-trimoxazole and amoxicillin (Table 4).

**Table 3:** The association of febrile illnesses with sex, age and season.

		Odds Ratio	95%CI	P-value
**Malaria**	Male	1.00		
	Female	0.90	0.55–1.46	0.661
	< 5 years	1.00		
	≥ 5 years	1.09	0.66–1.79	0.744
	Wet season	1.00		
	Dry season	1.53	0.92–2.53	0.097
**Presumptive leptospirosis**	Male	1.00		
	Female	1.91	0.98–3.75	0.059
	< 5 years	1.00		
	≥ 5 years	1.87	0.95–3.68	0.072
	Wet season	1.00		
	Dry season	0.42	0.21–0.86	0.017
**Confirmed leptospirosis**	Male	1.00		
	Female	0.37	0.15–0.88	0.025
	<5 years	1.00		
	≥ 5 years	1.29	0.56–2.94	0.546
	Wet season	1.00		
	Dry season	0.58	0.25–1.32	0.193
**Presumptive brucellosis**	Male	1.00		
	Female	0.97	0.42–2.21	0.939
	< 5 years	1.00		
	≥ 5 years	3.84	1.48–10.01	0.006
	Wet season	1.00		
	Dry season	0.40	0.16–1.01	0.051
**Probable prior brucellosis**	Male	1.00		
	Female	2.51	1.24–5.07	0.010
	< 5 years	1.00		
	≥ 5 years	2.78	1.37–5.66	0.005
	Wet season	1.00		
	Dry season	0.57	0.28–1.15	0.117
***B*. *melitensis***	Male	1.00		
	Female	1.78	0.96–3.31	0.067
	< 5 years	1.00		
	≥ 5 years	8.02	3.76–17.11	<0.001
	Wet season	1.00		
	Dry season	0.60	0.31–1.13	0.112
***B*. *abortus***	Male	1.00		
	Female	1.04	0.46–2.33	0.928
	< 5 years	1.00		
	≥ 5 years	1.25	0.55–2.84	0.598
	Wet season	1.00		
	Dry season	0.42	0.17–1.102	0.054
**Urinary Tract Infections**	Male	1.00		
	Female	3.02	1.70–5.34	<0.001
	< 5 years	1.00		
	≥ 5 years	1.12	0.66–1.95	0.685
	Wet season	1.00		
	Dry season	0.86	0.49–1.49	0.585
**Presumptive typhoid fever**	Male	1.00		
	Female	2.38	1.14–4.98	0.021
	< 5 years	1.00		
	≥ 5 years	2.20	1.06–4.57	0.034
	Wet season	1.00		
	Dry season	1.11	0.54–2.28	0.781

**Table 4:** The commonly prescribed antibacterial drugs.

	Amoxicillin n/N (%)	Ampicillin n/N (%)	Co-trimoxazole n/N (%)	Crystapen n/N (%)	Gentamycin n/N (%)	Ciprofloxacin n/N (%)	Erythromycin n/N (%)	Other antibiotics ^ξ^ n/N (%)
Malaria	6/34 (17.6)	0	26/34 (76.5)	2/34 (5.9)	0	0	1/34 (2.9)	0
Presumptive leptospirosis	9/35 (25.7)	1/35 (2.9)	12/35 (34.3)	5/35 (14.3)	1/35 (2.9)	4/35 (11.4)	3/35 (8.6)	5/35 (14.3)
Confirmed leptospirosis	6/21 (28.6)	1/21 (4.8)	6/21 (28.6)	2/21 (9.5)	1/21 (4.8)	1/21 (4.8)	2/21 (9.5)	0
Presumptive brucellosis	5/25 (20.0)	0	11/25 (44.0)	3/25 (12.0)	1/25 (4.0)	1/25 (4.0)	1/25 (4.0)	6/25 (24.0)
Probable prior brucellosis	8/35 (22.9)	0	16/35 (45.7)	3/35 (8.6)	1/35 (2.9)	0	2/35 (5.7)	8/35 (22.9)
*B*. *abortus*	10/24 (41.7)	0	6/24 (25.0)	5/24 (20.8)	1/24 (4.2)	0	2/24 (8.3)	5/24 (20.8)
*B*. *melitensis*	9/51 (17.6)	3/51 (5.9)	19/51 (37.3)	3/51 (5.9)	3/51 (5.9)	2/51 (3.9)	4/51 (7.8)	10/51 (19.6)
Presumptive typhoid fever	6/31 (19.4)	1/31 (3.2)	12/31 (38.7)	4/31 (12.9)	2/31 (6.5)	2/31 (6.5)	1/31 (3.2)	7/31 (22.6)
Urinary tract infection	8/64 (12.5)	2/64 (3.1)	45/64 (70.3)	9/64 (14.1)	3/64 (4.7)	0	2/64 (3.1)	5/64 (7.8)

^ξ^ Chloramphenical, cloxacillin, and Tetracycline

### Brucellosis

Twenty six (7.0%) of all participants met the criteria for presumptive acute brucellosis due to *B*. *abortus*, including 13/189 (6.9%) males and 13/181 (7.2%) females. Presumptive acute brucellosis was more common among older children (≥ 5 years) than in children aged less than five years (OR = 3.84, 95% CI: 1.48–10.01). In addition, few cases occurred in dry season as compared to rainy season (OR = 0.40, 95% CI: 0.16–1.01). The prevalence of *B*. *abortus* and *B*. *melitensis* by tube agglutination test was 26 (7.0%) and 57 (15.4%), respectively. Similarly, most cases of *B*. *melitensis* occurred in children aged ≥ 5 years (OR = 8.02, 95% CI: 3.76–17.11) even though for *B*. *abortus* both age groups were equally affected (Table 3). Although there was no significant statistical difference, *B*. *melitensis* and *B*. *abortus* were detected more during the rainy season than during the dry season. Among 370 participants, 42 (11.4%) met the definition for probable prior brucellosis exposure including 13/189 (6.9%) males and 29/181 (16.0%) females. Among the patients with probable prior brucellosis exposure, 37 had only anti-*Brucella* IgM antibodies while 5 patients had both IgM and IgG. The prevalence of prior brucellosis, exposure was more in children aged above five years (OR = 2.78, 95% CI: 1.37–5.66). Similarly, despite the absence of statistical significance, few cases were detected during dry season as compared to rainy season (Table 3). The main diagnoses for patients with presumptive acute brucellosis, *B*. *melitensis* and *B*. *abortus* were UTIs followed by malaria and pneumonia (Table 2). The common prescribed drugs were antibiotics particularly co-trimoxazole, amoxicillin and other antibiotics (Table 4).

### Urinary tract infections

Of 370 enrolled participants, 9 children could not provide urine sample. Therefore, among the 361 patients who were screened, 69 (18.6%) had UTIs. Urinary tract infections were three times higher in females than in males (OR = 3.02, 95% CI: 1.70–5.34). The findings further show that both age groups were equally affected regardless of their age difference. The main diagnoses provided were malaria 11 (15.9%), pneumonia 16 (23.2%) and other diseases 4 (5.8%), (Table 2). Twelve (17.4%) of patients with UTIs were treated with anti-malarials while 64 (92.8%) were treated with antibacterials particularly co-trimoxazole 45 (70.3%), benzylpenicillin (crystapen) 9 (14.1%) and amoxicillin 8 (12.5%).

### Typhoid fever

This study shows that 38 (10.3%) of enrolled patients had presumptive typhoid fever. Presumptive typhoid fever cases were significantly higher in females than in males (OR = 2.38, 95% CI: 1.14–4.98). In addition, patients aged 5 years and above were represented with a high prevalence, 24 (14.6%) than children aged below 5 years, 14 (6.8%) (OR = 2.20, 95% CI: 1.06–4.57). It was further noted that the prevalence of presumptive typhoid fever was stable between the rainy and dry season. Thirteen (34.2%) of patients suspected having typhoid fever received anti-malarial drugs while 31 patients (81.6%) were treated with antibiotics. The commonly used antibiotics were co-trimoxazole and amoxicillin (Table 4).

### Multiple infections

A total of 146 children (39.5%) had a possibility of being infected with more than one disease and of these, 126 (34.1%) could have had two diseases. Among patients diagnosed with malaria, 13 (3.5%) were suspected having acute leptospirosis, 7 (1.9%) had confirmed leptospirosis while 13 (3.5%) could also be infected with typhoid fever, 12 (3.2%) with UTIs, and 9 (2.4%) with *B melitensis* (Table 5). Of the 69 patients with UTIs, 14 (3.8%) could be co-infected with *B*. *melitensis*, 8 (2.2%) with typhoid fever and 6 (1.6%) with leptospirosis. Also, 11 (3.0%) of patients with typhoid fever had *B*. *melitensis* while 9 (2.4%) had presumptive typhoid fever and leptospirosis. Furthermore, twenty (5.4%) of participants could have had more than two diseases. This included malaria, UTIs and *B*. *melitensis* 4 (1.1%); typhoid fever, leptospirosis and *B*. *melitensis* 4 (1.1%); and Malaria, UTIs and typhoid fever 3 (0.8%), Table 5.

**Table 5:** Summary for the occurrence of multiple infections (N = 370).

Disease	n (%)
Prevalence of multiple infections	146(39.5)
**Total number of patients with two diseases**	**126 (34.1)**
Malaria + UTI	12 (3.2)
Malaria + typhoid fever	13 (3.5)
Malaria + presumptive leptospirosis	13 (3.5)
Malaria + confirmed leptospirosis	7 (1.9)
Malaria + presumptive brucellosis	3 (0.8)
Malaria + probable prior *brucellosis*	7 (1.9)
Malaria + *B*. *abortus*	3 (0.8)
Malaria + *B melitensis*	9 (2.4)
UTI + typhoid fever	8 (2.2)
UTI + confirmed leptospirosis	6 (1.6)
UTI + *B*. *melitensis*	14 (3.8)
UTI + *B*. *abortus*	5 (1.4)
Typhoid + *B*. *melitensis*	11 (3.0)
Typhoid + *B*. *abortus*	5 (1.4)
Typhoid + confirmed leptospirosis	9 (2.4)
**Total number of patients with three diseases**	**20 (5.4)**
Malaria + UTI + typhoid fever	3 (0.8)
Malaria + UTI + *B*. *melitensis*	4 (1.1)
Malaria + typhoid + confirmed leptospirosis	2 (0.6)
UTI + Typhoid + *B*. *melitensis*	3 (0.8)
UTI + typhoid + confirmed leptospirosis	2 (0.6)
UTI + confirmed leptospirosis +*B*. *melitensis*	2 (0.6)
Typhoid + confirmed leptospirosis + *B*. *melitensis*	4 (1.1)

## Discussion

In the present study, we have demonstrated occurrence of non-malarial febrile illnesses due to bacterial infections in children in a malaria-endemic area of Tanzania. This study confirms the importance of some bacterial causes of fever including zoonotic diseases such as leptospirosis and brucellosis. The study also identified the presence multiple bacterial infections as well as co-infections between malaria and bacterial diseases. Furthermore, infected patients were characterized with similar symptoms and thus differential diagnosis could not be made in the absence of diagnostic tests.

This study has illustrated that besides malaria, diseases such leptospirosis, brucellosis, typhoid fever and urinary tract infections should be considered in the differential diagnosis of febrile children. It is well documented that in acute leptospirosis or brucellosis, IgM antibodies start appearing during the first week of illness which is usually followed by IgG in the second week [34, 35, 36]. Therefore, the detection of IgM antibodies from the acute serum in patients from the present study could be an indication of acute leptospirosis or brucellosis. However, these findings should be interpreted with caution since in leptospirosis IgM antibodies can persist for months and thus their detection could mean a past infection rather than an acute infection. A limitation of our study was its cross-sectional design, which did not allow a comparison of the acute serum with convalescent serum from the same patients which would have been useful in our interpretation of results. Contrary to results from previous studies in Tanzania and Asia, our findings have demonstrated a higher prevalence of presumptive and confirmed leptospirosis, brucellosis and UTI [2, 11, 13, 37, 38, 39].

Findings from the present study have indicated that leptospirosis and brucellosis occurred more during the rainy season than in the dry season. This could be due to the reason that the rains cause rodents to move to residential areas hence increasing contamination of the environment including surface water through shedding of *Leptospires* in urine. These findings are in line with the results obtained from previous studies which have shown a similar seasonal pattern [40, 41]. Similarly, extrinsic factors such as rainfall have contributed to high occurrence of brucellosis around this period. The increase is believed to be associated with parturition since many births occur during rainfall [42]. This is accompanied by extensive shedding of *Brucella* organisms among infected animals which increases environmental contamination with consequences of increasing risk of exposure to infection. In this study, confirmed leptospirosis was detected more often in male patients than in females, which is in agreement with the results of a study conducted in the Netherlands where 90% of reported leptospirosis cases occurred in male patients [43]. Kilosa district is an agricultural area with pastoral livestock keeping activities and traditionally, boys tend to assist their parents with farming activities and looking after animals hence increases risks of exposure. On the contrary, the high number of malaria cases in the dry season could be due to the existence of water bodies such as rice paddies, irrigation canals, ponds and streams in the study area. Our findings concur with observations from previous studies in Malawi and Kenya where cases of malaria were higher in the dry season than in the rainy season [44, 45].

Our results show that UTI cases were more frequent in females than in males. This could be due to differences in anatomical structure i.e. the shortness of the urethra in women with its close proximity to the anus makes it easier for bacteria to ascend in the urinary tract [46]. Furthermore, it was noted that majority of brucellosis cases, particularly *B*. *melitensis* occurred in children aged 5 years and above, similar to findings reported by Tanir *et al*. (2009) from Turkey and Majali and his colleague from Jordan [47, 48]. The reason for the high prevalence of brucellosis in this age group could be that older children are more likely involved in animal care hence they might be at increased risk of contact with infected animals. This study has shown that suspected typhoid fever cases were common in children aged 5 years and above. This finding agrees with observations made in Nigeria and Pakistan where typhoid fever was more common in children aged 5–9 years [49, 50]. A plausible explanation for high prevalence of typhoid fever in older children is the consumption of unsafe locally-made chilled drinks and ice creams at schools since these are school-aged children. In contrast to our findings, Breiman *et al*. (2012) documented highest rates of typhoid fever in both age groups i.e. children aged 5–9 years and 2–4 years old [51]. Moreover, the frequency of typhoid fever was greater among females than males, similar to previous studies in Pemba, Zanzibar and Nigeria [52, 53, 54].

The present findings revealed that some patients had previously been exposed to leptospirosis and brucellosis. It should be noted that several previous reports have indicated the possibility of chronic brucellosis based on detection of anti-*Brucella* IgG particularly in absence of IgM antibodies [55, 56, 57]. Proximity of the study area to wildlife reservoirs in the Mikumi National Park, presence of livestock as well as traditional habits of livestock keepers in the study area to consume raw milk may justify our finding of anti-Brucella IgG antibodies and the likelihood of chronic brucellosis.

Our findings indicate the possibility of multiple infections in a considerable number of patients. This included coexistence of malaria and bacterial infections and occurrence of dual or triple bacterial infections. This finding can be compared to the results of a recent study conducted in Tanzania where multiple diagnoses were observed in two thirds of the children [4]. This is also in agreement with a study conducted in Bangladesh where patients had double or triple infections [58]. The co-infection between malaria and other diseases poses a challenge in the management of patients particularly in areas where diagnostic facilities for non-malaria febrile illnesses are limited. Therefore, these evidences strengthen the need to consider causes of fever other than malaria in patients diagnosed with malaria. In addition, the diagnosis of febrile patients should include the possibility of multiple non-malaria febrile illnesses.

Despite being endemic in many developing countries, diseases such as leptospirosis, brucellosis and typhoid fever remain unrecognized, under-diagnosed or under-reported. This was clearly shown by the present study where the main diagnoses were malaria, UTI, pneumonia and upper respiratory tract infections while leptospirosis, brucellosis and typhoid fever were not diagnosed clinically in any patient. Our results show that diarrhea, vomiting, loss of appetite, joint pain, abdominal pain and headache were the common manifestations in malaria patients as well as in patients with bacterial infections. In addition, these symptoms are consistent with several possible diagnoses including viral diseases such as dengue fever and yellow fever [59, 60]. Similar findings were reported from a recent study in Bangladesh where diseases such as malaria, leptospirosis, typhoid fever and dengue fever had similar clinical presentation [58]. The presence of unremarkable and non-specific clinical presentations makes it difficult to diagnose these diseases based on clinical grounds alone. Therefore, reliable diagnostic tests are required to identify such illnesses.

Another finding of this study is the improper management of febrile patients. Patients received antibacterials or anti-malarials but not standard treatment for human brucellosis. In the treatment of human brucellosis, monotherapy and short-term antibiotic regimes are not considered [61]. The most common used antibiotic regimes include doxycycline in combination with rifampin or streptomycin or gentamicin [61]. On contrary, no participant received any of the regimes. Furthermore, delay in receiving the appropriate antimicrobial therapy might develop life-threatening symptoms which can be associated with poor outcomes [62]. This indicates that a timely and accurate therapy is essential in the management of bacterial febrile illnesses. Furthermore, our findings suggest that failure to identify non-malaria febrile illnesses can lead to the misuse of broad spectrum antibiotics which could predispose to development of antibiotic resistance [63].

### Strengths and limitations

The present study has reported the prevalence of some bacterial febrile illnesses in a district hospital which serves as a referral for primary health care facilities in the district and hence provides a good representation of the general population in the district. However, detection of IgM or IgG antibodies in acute serum without considering the convalescent serum could mislead the interpretation of results. A limitation of using a single serum sample in the demonstration of IgM/IgG antibodies is the absence of antibodies in early stages of the infections or the persistence of antibodies from the previous exposure. Another limitation is that we did not conduct culture for typhoid fever and urinary tract infections. However, despite the design of the present study being cross-sectional and some diagnostic tests used were not 100% sensitive or specific, useful information has been gleaned from this study. This information may help the clinicians, policy makers and national disease control programs in planning for better ways for management of such illnesses.

### Conclusion

Findings from the present study indicate that besides malaria, bacterial diseases such as UTIs and bacterial zoonoses (leptospirosis and brucellosis) are a prominent cause of acute febrile illnesses in Kilosa district, Tanzania. Despite their importance, these diseases are passed unrecognized or are being underreported. Clinical diagnosis is not sufficient to discriminate such illnesses and thus laboratory confirmation is essential. Our findings underline the need to improve strategies for the diagnosis and management of febrile conditions particularly in resource-limited settings and also to refine disease burden estimates of bacterial and other common causes of febrile illnesses in children.

## Supporting Information

S1 Checklist STROBE checklist. (DOC)

S1 Table Sensitivity and specificity of the commercial laboratory test kits used to test sera for bacterial causes of febrile illness in this study. (DOC)

## References

[1] D'Acremont V, Lengeler C, and Genton B (2010) Reduction in the proportion of fevers associated with Plasmodium falciparum parasitaemia in Africa: A systematic review. Malar J. 9: 240 10.1186/1475-2875-9-240 20727214 PMC2936918

[2] Capeding MR, Chua MN, Hadinegoro SR, Hussain IIHM, Nallusamy R, et al (2013) Dengue and other common causes of acute febrile illness in Asia: an active surveillance study in children. PLoS Negl Trop Dis. 7: e2331 10.1371/journal.pntd.0002331 23936565 PMC3723539

[3] Crump JA, Morrissey AB, Nicholson WL, Massung RF, Stoddard RA, et al (2013) Etiology of Severe Non-malaria Febrile Illness in Northern Tanzania: A Prospective Cohort Study. PLoS Negl Trop Dis. 7: e2324 10.1371/journal.pntd.0002324 23875053 PMC3715424

[4] D'Acremont V, Kilowoko M, Kyungu E, Philipina S, Sangu W, et al (2014) Beyond malaria-causes of fever in outpatient Tanzanian children. N Engl J Med. 370: 809–817. 10.1056/NEJMoa1214482 24571753

[5] Nadjm B, Mtove G, Amos B, Walker NF, Diefendal H, et al. (2012) Severe febrile illness in adult hospital admissions in Tanzania: a prospective study in an area of high malaria transmission. Trans R Soc Trop Med Hyg. 10.1016/j.trstmh.2012.08.006 23022040

[6] Ronald A (2002) The etiology of urinary tract infection: traditional and emerging pathogens. Am J Med. 113: 14–19. 10.1016/s0002-9343(02)01055-0 12113867

[7] Mtove G, Amos B, von Seidlein L, Hendriksen I, Mwambuli A, et al (2010) Invasive salmonellosis among children admitted to a rural Tanzanian hospital and a comparison with previous studies. PLoS One. 5: e9244 10.1371/journal.pone.0009244 20168998 PMC2821934

[8] Goris MGA and Hartskeerl RA (2013) Leptospirosis serodiagnosis by the microscopic agglutination test. Curr Protoc Microbiol: 12E. 5.1-12E. 5.18. 10.1002/9780471729259.mc12e05s32 24510846

[9] Corbel MJ, Brucellosis in humans and animals. 2006: World Health Organization.

[10] Chipwaza B, Mugasa JP, Mayumana I, Amuri M, Makungu C, et al (2014) Community Knowledge and Attitudes and Health Workers' Practices regarding Non-malaria Febrile Illnesses in Eastern Tanzania. PLoS Negl Trop Dis. 8: e2896 10.1371/journal.pntd.0002896 24852787 PMC4031176

[11] Biggs HM, Hertz JT, Munishi OM, Galloway RL, Marks F, et al (2013) Estimating Leptospirosis Incidence Using Hospital-Based Surveillance and a Population-Based Health Care Utilization Survey in Tanzania. PLoS Negl Trop Dis. 7: e2589 10.1371/journal.pntd.0002589 24340122 PMC3855074

[12] Biggs HM, Bui DM, Galloway RL, Stoddard RA, Shadomy SV, et al (2011) Leptospirosis among hospitalized febrile patients in northern Tanzania. Am J Trop Med Hyg. 85: 275 10.4269/ajtmh.2011.11-0176 21813847 PMC3144825

[13] Bouley AJ, Biggs HM, Stoddard RA, Morrissey AB, Bartlett JA, et al (2012) Brucellosis among hospitalized febrile patients in Northern Tanzania. Am J Trop Med Hyg. 87: 1105–1111. 10.4269/ajtmh.2012.12-0327 23091197 PMC3516083

[14] NBS (2013) 2012 population and housing census Tanzania National Bureau of Statistics

[15] NBS (2013) 2012 population and housing census Population Distribution by Age and Sex. Tanzania National Bureau of Statistics.

[16] MRCO (2006) Morogoro region socio-economic profile Morogoro Regional Commissioner's Office.

[17] KDC (2008) Council Annual Report—2008. Kilosa District Council.

[18] MOH (2003) Tanzania National Health Policy. Ministry of Health.

[19] Wort U, Hastings I, Mutabingwa TK, and Brabin B (2006) The impact of endemic and epidemic malaria on the risk of stillbirth in two areas of Tanzania with different malaria transmission patterns. Malar J. 5: 89 17044915 10.1186/1475-2875-5-89 PMC1624843

[20] NBS (2008) AIDS and malaria indicator survey 2007–08 Dar es Salaam: Tanzania Commission for AIDS, ZAC, National Bureau of Statistics, Office of the Chief Government Statistician and ORC Macro.

[21] NBS (2011) Tanzania Demographic and Health Survey-2010 National Bureau of Statistics (Tanzania) and ICF Macro:Tanzania Demographic and Health Survey.

[22] Mwisongo AJ, Kisoka WJ, Mubyazi GM, Malebo H, Senkoro KP, et al (2001) Major health problems in some selected districts of Tanzania. Tanzan J Health Res. 3: 10–14.

[23] MOHSW (2011) Sentinel Panel of Districts Ministry of Health and Social Welfare, the National Bureau of Statistics, National Institute for Medical Research & Ifakara Health Institute.

[24] DMO (2006) Kilosa District Annual Report, Kilosa District Medical Office.

[25] DMO (2013) Kilosa District Annual Report, Kilosa District Medical Office.

[26] Naing L, Winn T, and Rusli BN (2006) Practical issues in calculating the sample size for prevalence studies. Arch Orofac Sci. 1: 9–14. 17694640

[27] Greenwood B and Armstrong JRM (1991) Comparison of two simple methods for determining malaria parasite density. Trans R Soc Trop Med Hyg. 85: 186–188. 1887466 10.1016/0035-9203(91)90015-q

[28] Mgode GF, Katakweba AS, Mhamphi GG, Fwalo F, Bahari M, et al (2014) Prevalence of leptospirosis and toxoplasmosis: a study of rodents and shrews in cultivated and fallow land, Morogoro rural district, Tanzania. Tanzan J Health Res. 16. 10.4314/thrb.v16i3.11 26867284

[29] Goris MGA, Leeflang MMG, Loden M, Wagenaar JFP, Klatser PR, et al (2013) Prospective evaluation of three rapid diagnostic tests for diagnosis of human leptospirosis. PLoS Negl Trop Dis. 7: e2290 10.1371/journal.pntd.0002290 23875034 PMC3708816

[30] Honarmand HR and Eshraghi SS (2011) Detection of Leptospires serogroups, Which Are Common Causes of Human Acute Leptospirosis in Guilan, Northern Iran. Iran J Public Health. 40: 107 23113063 PMC3481722

[31] Nabukenya I, Kaddu-Mulindwa D, and Nasinyama G (2013) Survey of Brucella infection and malaria among Abattoir workers in Kampala and Mbarara Districts, Uganda. BMC Public Health. 13: 901 10.1186/1471-2458-13-901 24079448 PMC3852410

[32] Ley B, Mtove G, Thriemer K, Amos B, von Seidlein L, et al (2010) Evaluation of the Widal tube agglutination test for the diagnosis of typhoid fever among children admitted to a rural hospital in Tanzania and a comparison with previous studies. BMC Infect Dis. 10: 180 10.1186/1471-2334-10-180 20565990 PMC2898821

[33] Roberts KB (2011) Urinary tract infection: clinical practice guideline for the diagnosis and management of the initial UTI in febrile infants and children 2 to 24 months. Pediatr. 128: 595–610. 10.1542/peds.2011-1330 21873693

[34] Budihal SV and Perwez K (2014) Leptospirosis Diagnosis: Competency of Various Laboratory Tests. J Clin Diagn Res. 8: 199 10.7860/JCDR/2014/6987.4161 24596774 PMC3939550

[35] Goris MGA, Boer KR, Boumanan-Strijker M, Hartskeerl R, Lucas C, et al (2011) Serological laboratory tests for diagnosis of human leptospirosis in patients presenting with clinical symptoms The Cochrane Library.

[36] Al Dahouk S and Nöckler K (2011) Implications of laboratory diagnosis on brucellosis therapy. Expert Rev Anti Infect Ther. 9: 833–845. 10.1586/eri.11.55 21810055

[37] Punjabi NH, Taylor WRJ, Murphy GS, Purwaningsih S, Picarima H, et al (2012) Etiology of Acute, Non-Malaria, Febrile Illnesses in Jayapura, Northeastern Papua, Indonesia. Am J Trop Med Hyg. 86: 46–51. 10.4269/ajtmh.2012.10-0497 22232450 PMC3247108

[38] Ron-Román J, Ron-Garrido L, Abatih E, Celi-Erazo M, Vizcaı´no-Ordo´n˜ ez L, et al (2014) Human Brucellosis in Northwest Ecuador: Typifying Brucella spp., Seroprevalence, and Associated Risk Factors. Vector Borne Zoonotic Dis. 14: 124–133. 10.1089/vbz.2012.1191 24410144

[39] Swai ES and Schoonman L (2009) Human Brucellosis: Seroprevalence and Risk Factors Related to High Risk Occupational Groups in Tanga Municipality, Tanzania. Zoonoses Public Health. 56: 183–187. 10.1111/j.1863-2378.2008.01175.x 18811674

[40] Reller ME, Wunder EA Jr, Miles JJ, Flom JE, Mayorga O, et al (2014) Unsuspected Leptospirosis Is a Cause of Acute Febrile Illness in Nicaragua. PLoS Negl Trop Dis. 8: e2941 10.1371/journal.pntd.0002941 25058149 PMC4109853

[41] Goris MGA, Kikken V, Straetemans M, Alba S, Goeijenbier M, et al (2013) Towards the Burden of Human Leptospirosis: Duration of Acute Illness and Occurrence of Post-Leptospirosis Symptoms of Patients in The Netherlands. PLoS One. 8: e76549 10.1371/journal.pone.0076549 24098528 PMC3789694

[42] Mwebe R, Nakavuma J, and Moriyón I (2011) Brucellosis seroprevalence in livestock in Uganda from 1998 to 2008: a retrospective study. Trop Anim Health Prod. 43: 603–608. 10.1007/s11250-010-9739-3 21082245

[43] Goris MGA, Boer KR, Duarte TATE, Kliffen SJ, and Hartskeerl RA (2013) Human leptospirosis trends, the netherlands, 1925–2008. Emerg Infect Dis. 19: 371 10.3201/eid1903.111260 23622144 PMC3647640

[44] Townes LR, Mwandama D, Mathanga D, and Wilson ML (2013) Elevated dry-season malaria prevalence associated with fine-scale spatial patterns of environmental risk: a case-control study of children in rural Malawi. Malar J. 12: 407 10.1186/1475-2875-12-407 24206777 PMC3833815

[45] Idris ZM, Chim WC, Chang SD, Masatsugu K, Isao T, et al (2014) Geographic and seasonal variation in malaria prevalence on islands in Lake Victoria (western Kenya): results from three cross sectional studies. Malar J. 13: P61.

[46] Minardi D, D’Anzeo G, Cantoro D, Conti A, and Muzzonigro G (2011) Urinary tract infections in women: etiology and treatment options. Int J Gen Med. 4: 333 10.2147/IJGM.S11767 21674026 PMC3108201

[47] Al-Majali AM and Shorman M (2009) Childhood brucellosis in Jordan: prevalence and analysis of risk factors. Int J Infect Dis. 13: 196–200. 10.1016/j.ijid.2008.06.012 18786846

[48] Tanir G, Tufekci SB, and Tuygun N (2009) Presentation, complications, and treatment outcome of brucellosis in Turkish children. Pediatr Int. 51: 114–119. 10.1111/j.1442-200X.2008.02661.x 19371290

[49] Rabasa AI, Mava Y, Pius S, Timothy SY, and Baba UA (2013) Typhoid fever in children: Clinical presentation and risk factors. Niger J Paediatr. 40: 60–63.

[50] Siddiqui FJ, Rabbani F, Hasan R, Nizami SQ, and Bhutta ZA (2006) Typhoid fever in children: some epidemiological considerations from Karachi, Pakistan. Int J Infect Dis. 10: 215–222. 16431148 10.1016/j.ijid.2005.03.010

[51] Breiman RF, Cosmas L, Njuguna H, Audi A, Olack B, et al (2012) Population-based incidence of typhoid fever in an urban informal settlement and a rural area in Kenya: implications for typhoid vaccine use in Africa. PLoS One. 7: e29119 10.1371/journal.pone.0029119 22276105 PMC3261857

[52] Chijioke-Osuji CC and Duru FC (2014) Prevalence of antibody titre in healthy individual and enteric fever patients in Owerri, Nigeria. J Public Health Epidemiol. 8: 192–196.

[53] Thriemer K, Ley BB, Ame SS, Deen JL, Pak GD, et al (2012) Clinical and epidemiological features of typhoid fever in Pemba, Zanzibar: assessment of the performance of the WHO case definitions. PLoS One. 7: e51823 10.1371/journal.pone.0051823 23284780 PMC3527440

[54] Itah AY and Uweh EE (2005) Bacteria isolated from blood, stool and urine of typhoid patients in a developing country. Southeast Asian J Trop Med Public Health. 36: 673–677. 16124436

[55] Ahmad B, Jamil S, Bashir S, Bilal M, Hassan S, et al (2014) Incidence of Brucella Abortus and Brucella Melitensis In Peshawar And Identification of Active and Passive Infection. Life Sci J. 11.

[56] Diaz R, Casanova A, Ariza J, and Moriyon I (2011) The Rose Bengal test in human brucellosis: a neglected test for the diagnosis of a neglected disease. PLoS Negl Trop Dis. 5: e950 10.1371/journal.pntd.0000950 21526218 PMC3079581

[57] Aniyappanavar D, Prasad SR, Tanveer KM, and Rao S (2013) Brucella infections in high-risk population and in patients hospitalized for fever: A serological study at Kolar, Karnataka. Ann Trop Med Public Health. 6: 549.

[58] Swoboda P, Fuehrer H-P, Ley B, Starzengruber P, Ley-Thriemer K, et al (2014) Evidence of a Major Reservoir of Non-Malarial Febrile Diseases in Malaria-Endemic Regions of Bangladesh. Am J Trop Med Hyg. 90: 377–382. 10.4269/ajtmh.13-0487 24420774 PMC3919252

[59] Rowe EK, Leo Y-S, Wong JGX, Thein T-L, Gan VC, et al (2014) Challenges in Dengue Fever in the Elderly: Atypical Presentation and Risk of Severe Dengue and Hospita-Acquired Infection. PLoS Negl Trop Dis. 8: e2777 10.1371/journal.pntd.0002777 24699282 PMC3974675

[60] Thomas RE, Lorenzetti DL, Spragins W, Jackson D, and Williamson T (2012) The safety of yellow fever vaccine 17D or 17DD in children, pregnant women, HIV+ individuals, and older persons: systematic review. Am J Trop Med Hyg. 86: 359–372. 10.4269/ajtmh.2012.11-0525 22302874 PMC3269291

[61] Ariza J, Bosilkovski M, Cascio A, Colmenero JD, Corbel MJ, et al (2007) Perspectives for the treatment of brucellosis in the 21st century: the Ioannina recommendations. PLoS Med. 4: e317 18162038 10.1371/journal.pmed.0040317 PMC2222927

[62] Cascio A, De Caridi G, Lentini S, Benedetto F, Stilo F, et al (2012) Involvement of the Aorta in Brucellosis: The Forgotten, Life-Threatening Complication. A Systematic Review. Vector Borne Zoonotic Dis. 12: 827–840. 10.1089/vbz.2012.0965 22994597

[63] Lubell Y, Turner P, Ashley EA, and White NJ (2011) Susceptibility of bacterial isolates from community-acquired infections in sub-Saharan Africa and Asia to macrolide antibiotics. Trop Med Int Health. 16: 1192–1205. 10.1111/j.1365-3156.2011.02837.x 21740488

