# Supplementary material for: Prevalence of Bacterial Febrile Illnesses in Children in Kilosa District, Tanzania
Source: PLoS Negl Trop Dis. 2015 May 8;9(5):e0003750. doi: 10.1371/journal.pntd.0003750 (PMC4425467; doi:10.1371/journal.pntd.0003750)
Supplement: S1 Table — (DOC) [file pntd.0003750.s002.doc]

**Table S1.** Sensitivity and specificity of the commercial laboratory test kits used to test sera for bacterial causes of febrile illness in this study

| **Kit (Manufacturer)** | **Sensitivity** | **Specificity** | **Source of information** |
| --- | --- | --- | --- |
| **NovaLisa *Brucella* IgM ELISA** | >95% | >95% | Product information (available at <http://www.novatec-id.com/fileadmin/user_upload/Product_Insert/BRUM0050engl_dt_fr_it_es_port-23022011.pdf> and additional information provided by manufacturer on request |
| **NovaLisa *Brucella* IgG ELISA** | >95 % | >95 % | <http://www.novatec-id.com/fileadmin/user_upload/Product_Insert/BRUG0050engl_dt_fr_it_es_port-23022011.pdf> |
| **Serion ELISA Classic *Leptospira* IgM** | 97 % | 96 % | <http://www.virion-serion.de/download/gebrauchsanweisung/leptospira/IFU-Leptospira-DE-EN-FR-IT-RU.pdf> |
| **Serion ELISA Classic Leptospira IgG** | n.d.* | 97% | <http://www.virion-serion.de/download/gebrauchsanweisung/leptospira/IFU-Leptospira-DE-EN-FR-IT-RU.pdf> |

^*^The sensitivity of the ELISA *Leptospira* IgG test based upon this study is not particularly helpful or meaningful as MAT and IgG immunoassays correlate poorly. False-positive results in the ELISA IgG may be true-positive, since *Leptospira*-infections may be subclinically and persisting IgG antibodies may be undetectable in MAT. Furthermore, in early *Leptospira*-infections an IgG response may be missing. For this reason, no statement is given for the sensitivity of SERION classic *Leptospira* IgG ELISA.
